# Associations Between Adverse Childhood Experiences and Prenatal Mental Health in the French EDEN Cohort: Cumulative, Person-Centered, and Dimensional Approaches

**DOI:** 10.1155/da/1295206

**Published:** 2025-05-06

**Authors:** Sara Avendano, Muriel Tafflet, Cedric Galéra, Laetitia Davidovic, Barbara Heude, Judith van der Waerden

**Affiliations:** ^1^Social Epidemiology, Mental Health and Addiction Team (ESSMA), Pierre Louis Institute of Epidemiology and Public Health, INSERM, Sorbonne University, Paris 75012, France; ^2^Obstetric, Perinatal, Paediatric Life Course Epidemiology (OPPaLE), Center for Research in Epidemiology and Statistics, INSERM, INRAE, Paris Cité University and Sorbonne University Paris Nord, Paris 75004, France; ^3^Epidemiology, Development and Prevention of Mental Health Problems using a Life Span Perspective (Healthy), Bordeaux Population Health Research Center, INSERM U1219, Bordeaux University, Bordeaux 33000, France; ^4^Charles Perrens Hospital, Bordeaux 33076, France; ^5^Microbiota, Immunity, and Neurodevelopment (MINDev), Institute of Molecular and Cellular Pharmacology, CNRS UMR7275, INSERM U1318, Université Côte d'Azur, Valbonne 06560, France

**Keywords:** adverse childhood experiences, anxious symptoms, depressive symptoms, latent class analysis, pregnancy, threat and deprivation

## Abstract

**Background:** Adverse childhood experiences (ACEs) may negatively affect prenatal mental health. However, the use of a cumulative ACEs score may obscure the identification of which specific types of adversity are most strongly associated with unfavorable mental health outcomes.

**Aim:** This study aims to evaluate the association between ACEs and prenatal symptoms of depression and anxiety using a cumulative score, a person-centered approach, and the dimensional model of adversity and psychopathology (DMAP).

**Methods:** Data were collected from 1887 pregnant women in the French Etude des Déterminants du développement et de la santé de l'ENfant (EDEN) cohort. To operationalize our exposure, we calculated a cumulative ACE score, threat and deprivation scores, and conducted latent class analysis (LCA). Depressive and anxious symptoms were assessed with the Center for Epidemiologic Studies-Depression Scale (CES-D) and the State-Trait Anxiety Inventory state subscale (STAI-S) questionnaires, using cutoffs of 16 and 38 indicating high symptoms. Participants were categorized into four outcome groups: (1) no symptoms, (2) high depressive symptoms only, (3) high anxious symptoms only, and (4) comorbid high symptoms. Multinomial regressions were performed.

**Results:** LCA identified three ACE classes: low-risk, family discordance, and multidimensional adversity. Women reporting two or more ACEs had higher odds of depressive and comorbid symptoms, compared to those with zero ACEs. Compared to the low-risk class, women in the family discordance class had increased odds of high depressive symptoms (adjusted odds ratios [aOR] 95% confidence interval [CI] = 1.80 [1.33, 2.56]) and comorbid high symptoms (aOR [95% CI] = 2.04 [1.43, 2.89]). Threat experiences were significantly linked to high depressive symptoms (aOR [95% CI] = 1.48 [1.22, 1.79]) and comorbid high symptoms (aOR [95% CI] = 1.53 [1.25, 1.87]).

**Conclusion:** Using the DMAP and LCA approaches, we found that ACEs related to the familial environment and relationships during childhood were most strongly associated with prenatal high depressive and comorbid symptoms. This highlights the importance of operationalizing ACEs beyond a cumulative score to better capture their role in the development of prenatal mental health difficulties.

## 1. Introduction

Adverse childhood experiences (ACEs) are potentially traumatic events occurring before the age of 18. These events encompass a wide range of exposures, including experiencing or witnessing physical, emotional, or sexual abuse, experiencing physical or emotional neglect, and growing up in a dysfunctional household characterized by issues such as substance abuse, mental health problems, parental separation, or the incarceration of a family member [1]. Additionally, adverse events within the child's community, such as racism, living in an unsafe neighborhood, living in poverty, and bullying, can also be considered ACEs [2, 3].

Adversity during critical windows of heightened brain plasticity may have enduring negative effects on neurodevelopment [4] increasing the risk of mental health difficulties later in life, which may persist into adulthood [5, 6]. These alterations include dysregulated stress responses (e.g., impairment of hypothalamic–pituitary–adrenal (HPA) axis feedback) [7], high inflammation states [8], imbalance between excitatory/inhibitory neurotransmission systems and microglial alteration [9], and delayed synaptic maturation [10]. Depending on their nature, ACEs may independently shape brain development with differential effects on mental health. According to the dimensional model of adversity and psychopathology (DMAP) framework [11], threat experiences (regarding interpersonal violence or that cause harm or threat of harm to the child) are expected to influence emotional reactivity and regulation as well as fear learning through neural changes in the hippocampus, amygdala, and ventromedial prefrontal cortex. On the other hand, deprivation experiences (involving the absence of expected caregiver inputs or social and cognitive stimulation) can lead to disruption in the synaptic pruning process and global decreases in gray matter volume [12, 13]. Additionally, these types of events can alter hippocampal–cortical connectivity, which may lead to worse executive functioning and language development [13].

Recently, attention has broadened to the potential mental health consequences that ACEs may have during pregnancy. The prenatal period is a sensitive time for both maternal and fetal well-being [14], marked by physiological, psychological, and sociofamilial changes, which increase vulnerability to mental health issues. Hence, assessing past experiences during pregnancy is crucial, as traumatic childhood memories may become particularly salient during this time. As women transition to parenthood and consider how they will care for their baby, these memories may resurface, influencing their mental health and emotional well-being [14, 15]. Indeed, a meta-analysis found that ACEs were positively associated with prenatal depressive and anxious symptoms [15]. Additionally, ACEs have been associated with certain unfavorable psychosocial factors in adulthood, such as low income and lack of stable relationships [16], which may further exacerbate prenatal depression and anxiety. Children of mothers with a history of ACEs may also be affected, as maternal mental health difficulties are linked to increased risk of emotional, behavioral, and developmental challenges in children [17–20].

The prevailing method for studying the consequences of ACEs consists of summing individual events into a cumulative score [1]. A dose–response relationship between the number of ACEs and the likelihood of experiencing prenatal depressive and anxious symptoms has been demonstrated [15]. However, this approach obscures the identification of which specific types of adversity might be most strongly associated with unfavorable outcomes [21] and limits understanding on the underlying mechanisms that link ACEs and mental health difficulties, thereby hindering the identification of specific targets for intervention [11]. To overcome these limitations, recent studies have focused on the person-centered approach using latent class analysis (LCA) [21, 22]. LCA consists of evaluating the co-occurrence of different events to identify combinations of adversities that might differentially confer an increased risk for the development of mental health problems. For instance, a recent study found that pregnant women belonging to the “childhood abuse/neglect” class reported higher levels of stress and mental health symptoms compared to women in the “low exposure” class, whereas women in a “childhood neglect only” class were similar to the “low exposure” class [23]. Other ACE research is based on the DMAP framework [11] mentioned earlier. Few studies have examined the relationship between dimensions of threat and deprivation experienced in childhood and mental health during pregnancy [24–26]. Yet, one of them showed that severe childhood threat, but not deprivation, was associated with more depressive symptoms during pregnancy [26].

A limited number of studies in the broader literature have compared different operationalizations of ACEs in relation to mental health outcomes [27–29], showing that assessing ACEs beyond a cumulative score provides a more nuanced understanding of their health consequences. However, to our knowledge, none of these comparative studies has focused on pregnant populations. In the French context, the study of ACEs and mental health has primarily examined adolescents [30, 31] and young adults [32], relying mostly on cumulative scores. Meanwhile, prenatal psychosocial factors [33] have received comparatively less attention. Understanding the role of ACEs in contributing to prenatal depression and anxiety is essential for informing early interventions that support both maternal and child health. Given these gaps in the literature, the aim of this study was to evaluate the associations of ACEs with subsequent prenatal symptoms of depression and anxiety in the French EDEN cohort using the three outlined approaches (cumulative score, LCA, and DMAP). We hypothesized that there would be a dose–response relationship for the cumulative score, with higher ACE scores associated with a greater risk of prenatal depressive and anxious symptoms. For the LCA approach, we expected that the class identified as having the highest risk of childhood adversity would exhibit higher levels of depressive and anxiety symptoms during pregnancy compared to the class with the lowest risk. Finally, in the DMAP approach, we hypothesized that participants who experienced threatening events would be at an increased risk of developing prenatal depressive and anxious symptoms. In addition, we hypothesized that ACEs would have a stronger effect on comorbid depressive and anxious symptoms than on depressive and anxious symptoms in isolation.

## 2. Materials and Methods

### 2.1. Study Sample

Data were obtained from the French cohort Etude des Déterminants du développement et de la santé de l'ENfant (EDEN), which examines the prenatal and early postnatal determinants of child health and development [34]. Between 2003 and 2006, pregnant women with a gestational age of less than 24 weeks were recruited at two study centers: the university hospitals of Nancy and Poitiers (France). Exclusion criteria were multiple pregnancies, prepregnancy diabetes, inability to speak French, and plans to change residence within the next 3 years. During pregnancy, biomedical data, sociodemographic characteristics, and information about the participants' childhood were collected through obstetrical records, self-administered questionnaires, and face-to-face interviews. A total of 2002 women accepted to participate, provided written consent, and were enrolled in the cohort. The longitudinal EDEN cohort received ethical approval from the Ethical Research Committee of Bicêtre Hospital and the French Data Protection Authority. Written informed consent was obtained from mothers at the time of enrollment for themselves and their newborns after delivery. All research was conducted per the Declaration of Helsinki.

#### 2.1.1. Current Study

**Figure 1:**
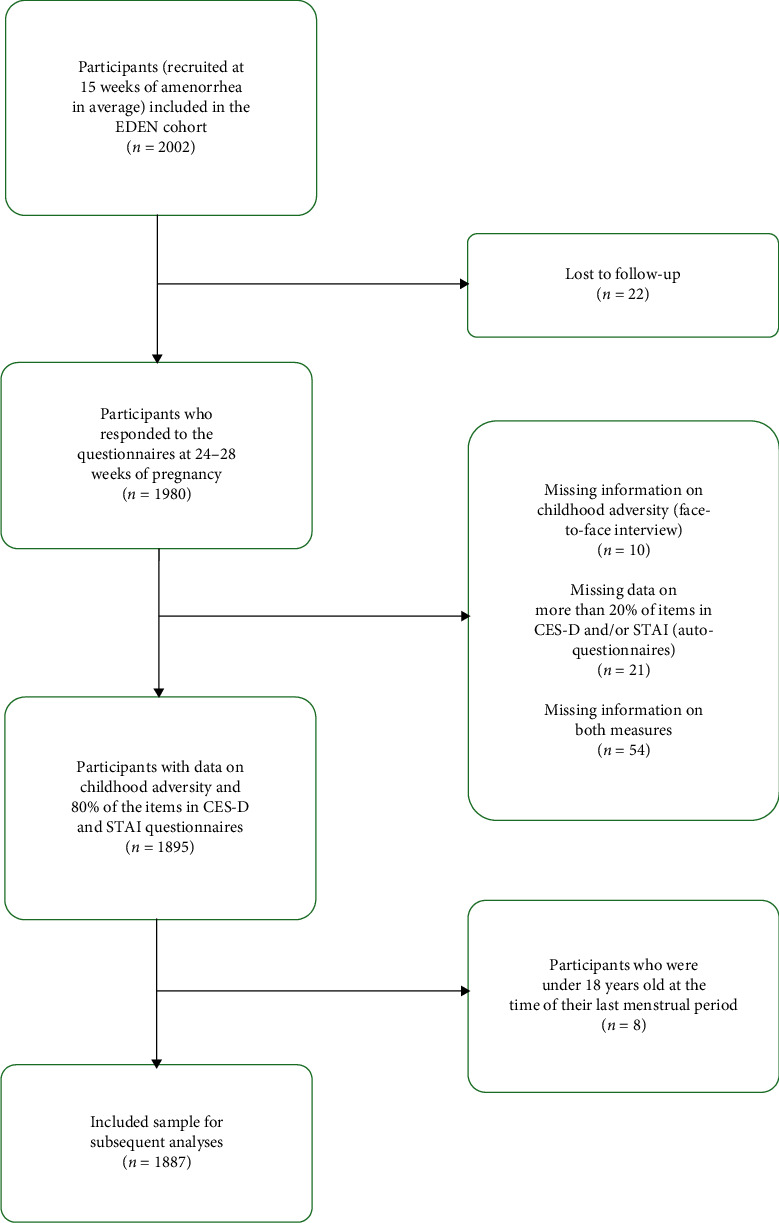
Flowchart of the inclusion of the study sample.

The current study presents primary analyses of the French mother–child cohort EDEN. For these analyses, we included participants who provided information on childhood adversity, completed at least 80% of the prenatal depression and anxiety symptoms questionnaires and were 18 years old or older. These inclusion criteria resulted in a sample of 1887 participants (Figure 1).

### 2.2. Measures

#### 2.2.1. Exposure: ACEs

Between 24 and 28 weeks of pregnancy, participants responded to the following questions in a face-to-face interview: “We are going to talk about situations that some people have experienced in their childhood or adolescence. Have you yourself (1) suffered material deprivation?, (2) had an out-of-home placement by the governmental assistance programs?, (3) been followed by child protective services?, (4) had a very serious conflict with one or both of your parents?, (5) witnessed severe tension or violence between your parents?, and (6) been subjected to abuse or repeated beatings?”. Events were coded as 1 if they had experienced them and as 0 if they had not. In addition, participants answered open-ended questions regarding their parents' occupations and their living situation when they were 14 years old. Despite that these items were not expressly asked in the ACE questionnaire, we were able to extract further information on (7) *parental divorce or separation*, (8) *death of a parent*, and (9) *low socioeconomic status* (*SES*) *of the household*. For the creation of this last variable, we used information on the occupation of the parents by using the ESeG classification [35]. We categorized the SES of the household into upper class, middle class, and working class [36], by selecting the parent with the highest class. For the creation of the binary indicator, we grouped the middle- and upper-class groups to be compared with the working-class group. Thus, in total, we had information on nine different indicators of childhood adversity, modeled as binary variables: material deprivation (yes/no), out of home placement (yes/no), child protective services (yes/no), serious conflict with parents (yes/no), severe tension or violence between parents (yes/no), subjected to abuse or beatings (yes/no), parental divorce or separation (yes/no), death of a parent (yes/no), and low SES of the household (yes/no).

#### 2.2.2. Outcome: Prenatal Symptoms of Depression and Anxiety

Prenatal symptoms of depression and anxiety were assessed through validated self-reported questionnaires between the 24 and 28 weeks of gestation. The Center for Epidemiologic Studies-Depression Scale (CES-D) is a 20-item questionnaire used for assessing depressive symptoms in the general population [37]. The CES-D has been translated and validated in the French population [38], and a recent study shows the validity of this scale during pregnancy [39]. Participants responded on a 4-point Likert scale for each item about depressive symptoms experienced in the past week. Scores range from 0 to 60, with higher scores indicating greater symptoms. In our sample, the CES-D score exhibited a Cronbach's alpha of 0.88 (95% CI 0.87, 0.89), indicative of the scale's internal consistency and reliability. To denote high depressive symptoms, a binary variable was created using a score of ≥16 as a cutoff [37], which is usually used for identification of individuals with clinical depression.

The State-Trait Anxiety Inventory (STAI) is an appropriate instrument for the study of anxiety in research and clinical settings [40], having been adapted and validated in the French population [41]. This scale measures two types of anxiety: state anxiety, which assesses the current emotional state, and trait anxiety, which evaluates relatively stable aspects of anxiety or how the participant “generally feels.” For this study, we used the STAI state subscale (STAI-S) items, which asked participants on a 4-point Likert scale about their feelings at the moment they were filling in the questionnaire. The Cronbach's alpha for STAI-S was of 0.91 (95% CI 0.90, 0.92), with scores ranging from 20 to 80. Since there is a lack of consensus in the literature regarding a cutoff for the French-adapted STAI-S, the threshold of ≥38 (80th percentile of our sample distribution) was used to dichotomize the variable, indicating high levels of prenatal anxiety. As part of sensitivity analyses, we re-ran the statistical models using a threshold of ≥40 which has been previously used internationally [42, 43].

Based on their binary classifications of depression and anxiety symptoms, participants were categorized into four groups: (1) no symptoms of depression or anxiety (CES-D < 16 and STAI-S < 38), (2) high depressive symptoms only (CES-D ≥ 16 and STAI-S < 38), (3) high anxious symptoms only (CES-D < 16 and STAI-S ≥ 38), and (4) comorbid high depressive and anxious symptoms (CES-D ≥ 16 and STAI-S ≥ 38).

#### 2.2.3. Covariates

First, with the help of a directed acyclic graph, potential confounders were considered if they were found in the literature to be associated with both exposure (ACEs) and outcome (prenatal depressive and anxious symptoms) and were available in the EDEN cohort. Since this study comprises several years between the exposure (which occurred at different points during childhood and adolescence—some before the age of 14 and others extending up to 18 years) and the outcome during pregnancy (≥18 years old), the considered confounders were limited to those retrospectively measured during the childhood and adolescence of the participants. Including adulthood covariates in the statistical model may lead to inclusion of potential mediators of the relationship between ACEs and prenatal mental health [44] leading to underestimation of the total effect. To determine inclusion in the final models, we then tested associations between these covariates, ACEs, and prenatal depressive and anxious symptoms. Covariates were retained if they were statistically significant (*p*  < 0.20) with either the exposure or the outcome or identified in the literature as major confounders. Thus, the following covariates were selected: age at pregnancy (years), migrant status (none, second generation, and first generation), primary education only (yes/no) as a proxy for socioeconomic status [45], being born preterm (yes/no) [46], and tobacco use of their mothers during pregnancy (yes/no) [47]. The study center was also included to increase precision.

### 2.3. Statistical Analyses

#### 2.3.1. Cumulative ACE Score

For the creation of the cumulative ACE score, we summed all the binary indicators of childhood adversity, leading to a score ranging from 0 to 9. Given the small proportion of participants in the highest ACEs count, the cumulative ACE score was winsorized, resulting in a categorical score whose values were 0, 1, 2, and ≥3.

#### 2.3.2. LCA

To identify different patterns of co-occurrence of ACEs, a person-centered approach was implemented by performing LCA. LCA is a form of finite mixture modeling useful for identification of mutually exclusive and exhaustive unobserved subgroups in a population, accounting for the patterns of response of the indicator variables [22, 48]. For construction of the latent classes, data were derived from participants who had information for at least one of the nine adversity indicators. Parameters were estimated by maximum likelihood using an expectation–maximization (EM) type procedure. Missing data are handled in this procedure, assuming data to be missing at random (MAR) [22]. To evaluate the convergence of each model and to avoid suboptimal local maxima of the likelihood function, 1000 random starting values were used for each model. Model selection was guided by fit indices including Akaike information criterion (AIC) and Bayesian information criterion (BIC) [49]. In addition, the bootstrap likelihood ratio test (BLRT) [50, 51] was used, where a low *p*-value indicates that the model with *k* classes has a better fit then the model with *k*-1 classes. Entropy close to 1.00 was also considered for precision of the model using the individual posterior probabilities of class membership. In addition to fit statistics, model selection was also dependent on theoretical meaningfulness, good distinction between the classes, and the percentage of the sample in each of the classes. PROC LCA version 1.3.2 was used in SAS version 9.4.

#### 2.3.3. Dimensional Approach

Using the same events as in the person-centered approach, we further evaluated the number of experiences related to dimensions of threat and deprivation. Exposure to threat experiences was assessed through the following indicators: subjected to abuse or beatings, conflict with parents, and severe tension/violence between parents. We summed these three measures and created an overall threat score ranging from 0 to 3. We assessed the dimension of deprivation through indicators of parental absence (parental separation and parental death), material deprivation, out-of-home placement, low SES household, and having had child protective services from the government. The sum of these indicators leads to the creation of an overall deprivation score ranging from 0 to 5. For the dimensional approach and following the methodology previously outlined by Stein et al. [52], the threat and deprivation scores were winsorized given the sparse observations in the upper tail, resulting in variables whose values were 0, 1, and ≥2.

#### 2.3.4. Descriptive Statistics

The prevalence of individual ACEs, sociodemographic characteristics, and prenatal depressive and anxious symptoms were described for the overall sample. Additionally, these characteristics were described by ACE score, latent class, and threat and deprivation scores. For the study sample description, Pearson's chi-squared test was used for categorical variables, Fisher's exact test was applied for continuous variables, and the Kruskal–Wallis rank sum test was employed for continuous variables that did not follow a Gaussian distribution.

#### 2.3.5. Multinomial Logistic Regressions

In a first exploratory step, we ran separate unadjusted multinomial logistic regressions using each individual ACE (presence vs. absence) as the independent variable and prenatal depressive and anxious symptoms as the outcome (reference group: “no symptoms of depression or anxiety"). To isolate the effect of each ACE, we then adjusted the model by including all other ACEs along with the confounders.

Subsequently, multinomial logistic regressions were used to study the relationship between ACEs and prenatal depressive and anxious symptoms comparing the three different approaches (overall cumulative ACE score, latent classes of adversity, and threat/deprivation scores). Prenatal symptoms of depression and anxiety were the outcome variable in all the performed regressions. Covariates that were found to be significantly related to both the outcome and the exposure were included in the adjusted models. In the model evaluating cumulative ACEs, we used the categorical score (0, 1, 2, ≥3) as the independent variable. When assessing the person-centered approach, latent classes of adversity (low risk for adversity, family discordance, and multidimensional adversity) served as the independent variable.

Regarding the dimensional approach, regressions were performed separately for threat and deprivation experiences as the independent variables. Then, to isolate the effect of each dimension, we included each variable in the other respective model as a covariate. To handle missing data, all analyses were performed on multiple imputed databases using the MICE package on R studio. Table S1 shows the number and percentage of missing data for each variable. All statistical analyses were conducted using R Studio (version 2023.12.0+369; R Core Team).

Sensitivity analyses were conducted using adjusted multinomial logistic regressions based on complete case analyses, which included only observations without missing values across all variables of interest. In addition, sensitivity analyses were conducted using an alternative cutoff of ≥40 for high anxious symptoms on the STAI-S.

## 3. Results

### 3.1. Characterization of ACE Latent Classes

**Figure 2:**
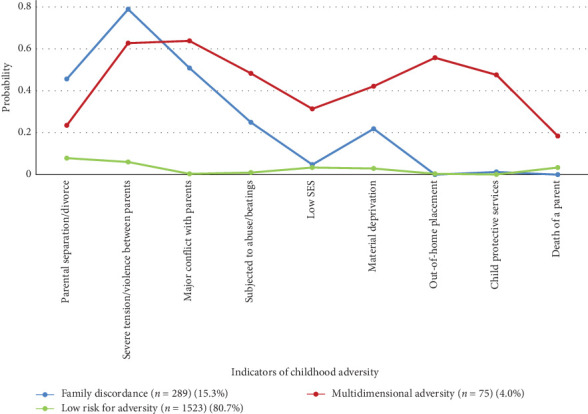
Three-class model of latent class analysis for the sample of the EDEN cohort.

Preliminary to our main analyses, we first characterized ACEs according to latent classes of adversity. Table S2 presents the fit statistics utilized for model selection from 1 to 4 latent classes. While the four-class solution had a lower AIC, lower BIC, and the BLRT indicated that this model had a better fit than the three-class model, it presented several small classes, which might be an issue for external generalization [53]. In addition, their theoretical meaningfulness was not adequate, given that only one indicator (*death of a parent*) determined one of the classes and its high entropy (0.88) might be due to overfitting of the model. Taken together, the three-class model was selected as the most suitable solution, presenting adequate fit statics with high entropy (0.84) and good theoretical interpretability.  Figure 2 provides a graphical representation of the patterns of the three classes of ACEs along with the item probabilities and class names.

Class 1, labeled *family discordance*, is characterized by having high probabilities of exposure to severe tension or violence between parents (79%) and moderate probabilities to major conflict with parents (51%). Respondents in this class had the highest probability of being exposed to parental separation/divorce (46%) compared to the other two classes. Class 2, labeled *multidimensional adversity*, is characterized by having moderate probabilities of conflict with parents and tension or violence between parents. This class was differentiated by the family discordance class by exhibiting moderate probabilities (42%–55%) for exposure to abuse or repeated beatings, material deprivation, out of home placement, and child protective services. Class 3, labeled *low risk for adversity*, is characterized by low probabilities (<10%) of exposure to any ACEs.

### 3.2. Descriptive Statistics

Table 1 shows the sample characteristics of the overall sample and by different adversity categories using the cumulative score, latent classes, and threat and deprivation scores. Women were on average 29 years old, 7% completed primary education only, almost 4% were first-generation migrants, and around 10% were second generation migrants. Among women, 6.5% were born preterm, and 10% of their mothers smoked during pregnancy. From the study sample, 61.7% of participants reported no ACE, while 16.9% reported experiencing one event, 10.7% reported two events, and 10.7% reported three or more events. Regarding the latent classes, 15.3% of the study sample were allocated to the *family discordance class*. The *multidimensional adversity class* represents 4%, and the *low risk for adversity class* represents 80.7%. Regarding specific dimensions of adverse experiences, 11.4% of the sample indicated having experienced two or more threat-related events, while 7.7% reported two or more deprivation-related events. When testing the sociodemographic characteristics of the participants, those who had higher scores of the cumulative ACEs, threat, and deprivation scores and those who were classified as having multidimensional adversity were younger, had lower educational attainment, and were more likely to be born preterm or have a mother who smoked during pregnancy, compared to those reporting no ACEs or classified as low-risk for adversity. Table S3 shows the statistical tests between the participants' characteristics and prenatal depressive and anxious symptoms.

The proportion of participants exposed to each of the ACEs is also shown in Table 1. In this sample, major tension between parents was the most reported ACE (21.0%), followed by parental divorce or separation (15.0%) and conflict with parents (12.0%). The least reported ACEs were out-of-home placement (3.0%) and child protective services (2.6%). Participants exhibiting only high depressive symptoms accounted for 15.0% of the sample, while those with only high anxiety symptoms represented 6.1%. Additionally, 13.0% of the participants experienced both high levels of depressive and anxious symptoms.

Compared to those who were included in the sample, excluded participants had fewer years of education and were more likely to have a first- or second-generation migration background (Table S4).

### 3.3. Cumulative ACE Score and Prenatal Depressive and Anxious Symptoms

Table 2 shows the results from the multinomial regressions examining the association between cumulative ACE score and prenatal *depressive and anxious symptoms*. Participants exposed to two ACEs had the highest odds of presenting prenatal high depressive symptoms only (adjusted odds ratios [aOR] 95% confidence interval [CI] = 2.82 [1.92, 4.16]) compared to those with no ACE exposure. Furthermore, the odds of comorbid high symptoms of depression and anxiety increased for participants exposed to two ACEs (aOR [95% CI] = 2.07 [1.35, 3.16]) and three or more ACEs (aOR [95% CI] = 2.31 [1.55, 3.46]). No significant associations were found between ACEs and prenatal high anxiety symptoms only.

### 3.4. Latent Classes of Adversity and Prenatal Depressive and Anxious Symptoms

Table 3 provides the results of multinomial linear regressions assessing the association between the three latent ACE classes and prenatal depressive and anxious symptoms. Compared to the low-risk class, the family discordance class presents higher odds of developing high depressive symptoms only (aOR [95% CI] = 2.01 [1.44, 2.81]) and comorbid high symptoms (aOR [95% CI] = 2.04 [1.44, 2.87]) during pregnancy when adjusting for confounders. Meanwhile, the participants in the multidimensional adversity class had a higher probability of developing comorbid high symptoms (OR [95% CI] = 2.59 [1.47, 4.58]); however, this association was no longer significant after adjustment. None of the exposed classes was associated with prenatal high anxious symptoms only. The indicator with the highest probability in the family discordance class was “Witnessing severe tension or violence between the parents.” This event was also the only one that remained significant with high depressive symptoms (aOR [95% CI] = 1.72 [1.20, 2.47]) in the adjusted multinomial regressions exploring the effects of individual ACEs (Table S5). Additionally, it was associated with comorbid high depressive and anxious symptoms (aOR [95% CI] = 1.78 [1.22, 2.60]).

### 3.5. Threat and Deprivation Experiences and Prenatal Depressive and Anxious Symptoms

For the dimensional approach, Table 4 shows the results from multinomial regressions evaluating the associations of threat and deprivation experiences on depressive and anxious symptoms during pregnancy. Threat experiences were significantly associated with high depressive symptoms only (aOR [95% CI] = 1.50 [1.23, 1.82]) and comorbid high symptoms (aOR [95% CI] = 1.57 [1.29, 1.92]) after adjustment for confounders and experiences of deprivation. No significant associations were observed between threat or deprivation experiences and high anxious symptoms only.

### 3.6. Sensitivity Analyses

Results from multinomial logistic regressions using complete cases are shown in Table S6. For theses analyses, 1547 observations were retained. The overall direction of these results was consistent with analyses using imputed datasets. Results from the multinomial logistic regressions using a cutoff of ≥40 for the STAI-S questionnaire are provided in the Table S7. Effect sizes were minimally attenuated, and the statistical significance of the results remained unchanged.

## 4. Discussion

The aim of our study was to evaluate the relationship between ACEs and maternal depressive and anxious symptoms during pregnancy in the French EDEN cohort using different approaches to operationalize ACEs. Other studies conducted in France [32] and other high-income countries have found that around 60%–70% of adults report at least 1 ACE [54]. In our sample, women reported fewer ACEs, with only 40% reporting at least one. This difference could be attributed to variations in how ACEs were measured and the specific types of ACEs assessed. In the EDEN cohort, we did not evaluate neglect or sexual abuse, potentially leading to an underestimation of the ACE prevalence in this sample. Despite this potential underestimation, and with the use of a cumulative ACE score, a LCA, and the DMAP approach, we found that women exposed during childhood to multiple adverse experiences, particularly to intrafamilial conflict or violence, were at higher risk for developing prenatal high depressive symptoms and comorbid high depressive and anxious symptoms, compared to unexposed women.

Our results align with previous research showing that ACEs are associated with an increased likelihood of depressive symptoms during pregnancy [15, 55] and comorbid symptoms of anxiety and depression [56, 57]. However, contrary to our hypothesis, we did not find an association between ACEs and the “high anxious symptoms only” group. While we identified a significant association with ACEs and the co-occurrence of high depressive and anxious symptoms, this may primarily reflect the influence of depressive symptoms, as the odds ratios were similar to the “high depressive symptoms only” group. A systematic review showed that childhood maltreatment was consistently associated with perinatal depression and post-traumatic stress disorder, but less so with anxiety [58], with fewer than half of the reviewed studies finding a significant positive association. The discrepancy may result from the simultaneous study of both depression and anxiety. For instance, in a clinical sample of depressed pregnant women, childhood trauma did not predict anxiety levels [59]. Conversely, others studies have shown that a higher number of ACEs is significantly associated with both anxiety and depressive disorders, though without accounting for comorbidity [60, 61]. Another possible explanation for our findings could be the method of anxiety measurement. The STAI-S may not fully capture prenatal anxiety, whereas pregnancy-specific anxiety measures might be more sensitive [62]. For instance, a cross-sectional study using a pregnancy-related anxiety questionnaire found that individuals with a high level of childhood adversity also had higher levels of anxiety [63]. Moreover, the lack of effect of ACEs on prenatal anxiety could also be influenced by the timing of assessment. Research suggests that maternal anxiety, as measured by the STAI, fluctuates throughout pregnancy, with higher scores typically observed in the third trimester [62]. In our study, however, anxious symptoms were assessed during the second trimester, which may have contributed to the absence of a significant effect. Future studies should further explore the relationship between ACEs and pregnancy related anxiety taking these considerations into account.

In line with previous studies and our hypotheses, using a cumulative ACEs score showed that individuals with two or more events were more likely to develop prenatal depressive symptoms and comorbid symptoms of anxiety and depression, compared to those with one or no ACE exposure, with a dose–response relationship for comorbid symptoms [64]. These findings indicate that the accumulation of multiple ACEs increases the risk of future prenatal mental health problems more than a single ACE does, supporting theories of allostatic load and toxic stress that may lead to neurodevelopmental alterations [65]. Cumulative exposure of ACEs may lead to repeated coping adaptations, which can activate multisystem (e.g., neurological and endocrinological) responses. Over time, this process contributes to the accumulation of physiological and psychological “wear-and-tear” on the body's systems [66, 67].

However, to better understand the role of ACEs in prenatal mental health, it is crucial to determine whether any additional ACE generally has a harmful effect or if specific patterns of ACEs exacerbate this risk. Indeed, when evaluating individual events, only witnessing severe tension or violence between parents was significantly associated with prenatal depressive symptoms and comorbid symptoms. This suggests that not all ACEs contribute equally to mental health outcomes [11, 68]. The LCA and the DMAP approaches allowed for a more detailed decomposition of childhood adversity in our sample.

LCA allowed us to identify three different classes of childhood adversity. Previous studies in the general population have typically identified between three and four latent ACE classes [21]. These usually include a low-risk class, a high-risk class with multiple adversities, and one or two classes characterized by specific ACEs with higher probabilities, in our study witnessing severe tension or violence between their parents and major conflict with their parents being the ones with the highest probabilities. Contrary to most studies that associate a multiple ACE class with the most severe negative mental health outcomes, individuals in our multidimensional class did not show significantly higher odds for depressive or anxiety symptoms, compared to the low risk for adversity class. This is likely because only 4% of our sample belonged to this multidimensional class, resulting in insufficient statistical power to detect a significant association. Additionally, certain types of ACEs that have been previously evaluated in other studies, such as sexual abuse, were not included in the current study. These have been shown to play a role in the development of prenatal depressive symptoms [69, 70].

Nevertheless, other studies have found that the multiple adversity class is not always the one conferring the highest risk [71, 72]. Specifically, our study found that the family discordance class was more strongly associated with high levels of depressive symptoms and comorbid symptoms of anxiety and depression during pregnancy. Research indicates that adults who were exposed to intimate partner violence during childhood show higher odds of depression [73] and low mental well-being [74]. Our findings indicate that the combination of different types of disagreements inside the family, and not only tension between the parents, may further increase the probability of high depressive symptoms and comorbid depressive and anxious symptoms. Although we are not aware of studies linking these types of events to prenatal mental health, Hemady et al. [71] found that mothers who experienced childhood emotional and physical abuse, and particularly those exposed to intrafamilial violence, were more likely to have babies with low birth weight compared to those belonging to a “low household dysfunction or abuse” class.

Using the DMAP approach, we observed that childhood threat experiences were associated with high depressive symptoms and comorbid depressive and anxiety symptoms during pregnancy. In contrast, deprivation experiences showed these associations only in unadjusted analyses. These findings align with previous work implementing the DMAP framework. For instance, Penner et al. [26] found that childhood abuse, but not neglect, was associated with greater depressive symptoms in pregnancy. Similar results were found in different socioeconomic and cultural settings. For example, one study in Pakistan found no relationship between childhood neglect and depressive symptoms in the perinatal period, whereas different forms of violence were associated with these symptoms [45]. Although research on the mental health impacts during pregnancy of the interplay between these adversities is limited, recent studies show that frequent interparental conflict is linked to parent–child conflict, which in turn increases the risk of depressive symptoms in both children [75] and adolescents [76]. It is possible that these early mental health problems continue into adulthood.

### 4.1. Research and Clinical Implications

To move beyond the use of a cumulative ACE score and gain a deeper understanding of how ACEs impact prenatal mental health, we employed both a theoretical (DMAP) and methodological approach (LCA) to capture adversity. Our findings showed that certain types and combinations of ACEs—particularly those related to the familial environment and relationships during childhood—were most strongly associated with prenatal mental health problems. Notably, for our study sample, both the DMAP framework and the LCA approach converged in highlighting the role of interparental tension or violence and major conflict with parents as key adverse experiences, corresponding to the concept of threat-related adversity. Despite their potential significance, these specific types of ACEs remain underexplored in relation to perinatal outcomes and warrant greater attention in future studies, including those focused on understanding on the underlying mechanisms linking ACEs to mental health difficulties.

Our results suggest that childhood conflictive relationships within the family may act as a potential risk factor for mental health difficulties during pregnancy. This insight could help identify specific leverage points for intervention. Screening for ACEs within antenatal care could enable healthcare providers to better identify pregnant women at higher risk of mental health issues due to a history of childhood adversity [77]. Identifying these women is crucial, as evidence suggests that women previously exposed to ACEs are also at increased risk of experiencing abuse during pregnancy [78], further compounding the mental health challenges they may face [23, 79]. In addition, a more comprehensive understanding of the role of ACEs during the prenatal period may provide insight into how to better support women with a high-risk profile through trauma-informed care, which might be beneficial for both the expectant mother and their children. A recent study evaluated the implementation of ACE screening in obstetric settings in the United States and found that such interventions offer practitioners a more holistic understanding of their patients' health [80].

### 4.2. Limitations and Strengths

Our results have to be interpreted within the limitations of our study. First, the childhood adversity questionnaire was not a validated instrument and did not explicitly measure certain important items, such as neglect and sexual abuse. However, these experiences might be partly captured through related items, such as out-of-home placement and requirement of child protective services. Additionally, we were able to extract further information on ACEs through questions about participants' living situations at age 14. Nevertheless, some ACEs occurring between the ages of 15 and 18 may not have been captured. In addition, our approach to proxying child poverty and SES with parental occupation may not have allowed us to identify the most deprived households. These factors might have led to an overall underreporting of the ACEs experienced.

Second, the questions were asked retrospectively, which could lead to memory bias. Retrospective measures of ACEs may be inconsistent over time, as they can be influenced by psychological distress linked to childhood experiences. For instance, participants who develop high levels of depression or psychological distress in adulthood may be more likely to report or remember ACEs [81, 82]. However, it has been reported that prospective and retrospective measures of ACEs have moderate agreement and are both associated with adult health outcomes [83]. Also, it has been found that changes in depressive symptoms are not associated with recall of ACEs [84]. Moreover, this type of questions may also be subjected to nonresponse and social desirability bias, particularly when asked in the presence of an interviewer, which can alter data accuracy.

Despite these limitations, our study also possesses multiple strengths. First, we were able to adjust for confounders that occurred during the childhood of the participants, ensuring that we did not include variables that may act as mediators in the relationship between ACEs and prenatal mental health. Second, we used validated instruments for depressive symptoms during pregnancy, enhancing the accuracy and reliability of our measurements. Another strength is the comparison of multiple approaches, as we considered methods that account for the theoretical aspects of adversity in childhood.

## 5. Conclusion

Our study underscores the importance of employing approaches what go beyond a cumulative score to comprehensively capture the complex interplay of ACEs on prenatal mental health outcomes. Our results show differential effects of latent classes of ACEs and threat and deprivation adversity dimensions on maternal depressive symptoms during pregnancy. In our sample, we consistently observed that threatful childhood events related to intrafamilial discord, such as conflict between parents or other family members, may pose a significant risk for developing mental health problems in adulthood, with a notable impact during pregnancy. The use of a more nuanced approach can provide complementary insights that may inform the development of targeted screening instruments and trauma-informed care in antenatal settings. Future research should explore the mechanisms underlying these relationships and how they may vary across other populations.

## Figures and Tables

**Table 1:** Description of characteristics of EDEN participants by cumulative ACE score, latent classes, and threat and deprivation scores.

		Cumulative ACE score					Latent classes			
Characteristic	Total^a^ (*n* = 1887)	0^a^ (*n* = 1165) (61.7%)	1^a^ (*n* = 319) (16.9%)	2^a^ (*n* = 201) (10.7%)	≥3^a^ (*n* = 202) (10.7%)	*p*-Value^b^	Low risk for adversity^a^ (*n* = 1523) (80.7%)	Multidimensional adversity^a^ (*n* = 75) (4.0%)	Family dysfunction^a^ (*n* = 289) (15.3%)	*p*-Value^b^
Age (in years)	29.55 (4.86)	29.9 (4.7)	29.2 (4.9)	29.3 (5.0)	28.6 (5.3)	0.002	29.7 (4.8)	28.5 (5.8)	29.1 (5.0)	0.053
Primary education only (yes)	130 (7.0%)	54 (4.7%)	16 (5.1%)	16 (8.2%)	44 (22%)	<0.001	76 (5.0%)	26 (35%)	28 (9.9%)	<0.001
Migration status						0.2				0.1
None	1593 (86%)	1008 (87%)	265 (85%)	161 (83%)	159 (81%)		1301 (86%)	57 (81%)	235 (83%)	
2nd generation	192 (10%)	106 (9.2%)	35 (11%)	23 (12%)	28 (14%)		147 (9.8%)	12 (17%)	33 (12%)	
1st generation	73 (3.9%)	40 (3.5%)	13 (4.2%)	10 (5.2%)	10 (5.1%)		57 (3.8%)	1 (1.4%)	15 (5.3%)	
Study center (Poitiers)	966 (51%)	624 (54%)	154 (48%)	88 (44%)	100 (50%)	0.039	797 (52%)	35 (47%)	134 (46%)	0.13
Personal history of prematurity (yes)	112 (6.5%)	60 (5.5%)	14 (4.8%)	17 (10%)	21 (12%)	<0.001	79 (5.6%)	9 (17%)	24 (9.6%)	0.001
Her mother smoked while being pregnant (yes)	179 (10%)	84 (7.5%)	36 (12%)	30 (17%)	29 (18%)	<0.001	123 (8.6%)	18 (33%)	38 (15%)	<0.001
Maternal mental health						<0.001				<0.001
No depressive or anxious symptoms	1212 (64%)	812 (70%)	199 (62%)	98 (49%)	103 (51%)		1026 (67%)	39 (52%)	147 (51%)	
High depressive symptoms only	287 (15%)	145 (12%)	53 (17%)	52 (26%)	37 (18%)		210 (14%)	13 (17%)	64 (22%)	
High anxious symptoms only	115 (6.1%)	68 (5.8%)	23 (7.2%)	13 (6.5%)	11 (5.4%)		94 (6.2%)	4 (5.3%)	17 (5.9%)	
Comorbid high symptoms	273 (14%)	140 (12%)	44 (14%)	38 (19%)	51 (25%)		193 (13%)	19 (25%)	61 (21%)	

Individual ACEs										

Material deprivation	154 (8.2%)									
Out of home placement	57 (3.0%)									
Child protective services	49 (2.6%)									
Conflict with parents	231 (12%)									
Tension between parents	400 (21%)									
Subjected to abuse/beatings	141 (7.5%)									
Parental separation/divorce	287 (15%)									
Death of a parent	65 (3.5%)									
Low SES of household	91 (4.9%)									

		Threat dimension score				Deprivation dimension score				
Characteristic	Total^a^ (*n* = 1887	0^a^ (*n* = 1397) (74.0%)	1^a^ (*n* = 275) (14.6%)	≥2^a^ (*n* = 215) (11.4%)	*p*-Value^b^	0^a^ (*n* = 1381) (73.2%)	1^a^ (*n* = 361) (19.1%)	≥2^a^ (*n* = 145) (7.7%)	*p*-Value^*b*^	
Age (in years)	29.55 (4.86)	29.6 (4.8)	29.5 (5.2)	29.2 (5.1)	0.6	29.9 (4.7)	28.7 (5.0)	28.4 (5.4)	<0.001	
Primary education only (yes)	130 (7.0%)	76 (5.5%)	25 (9.3%)	29 (14%)	<0.001	61 (4.5%)	30 (8.5%)	39 (28%)	<0.001	
Migration status					0.033				0.063	
None	1593 (86%)	1196 (87%)	230 (86%)	167 (79%)		1187 (87%)	297 (84%)	109 (79%)		
2nd generation	192 (10%)	129 (9.4%)	31 (12%)	32 (15%)		133 (9.7%)	37 (10%)	22 (16%)		
1st generation	73 (3.9%)	54 (3.9%)	7 (2.6%)	12 (5.7%)		47 (3.4%)	19 (5.4%)	7 (5.1%)		
Study center (Poitiers)	966 (51%)	748 (54%)	123 (45%)	95 (44%)	0.003	711 (51%)	186 (52%)	69 (48%)	0.7	
Personal history of prematurity (yes)	112 (6.5%)	74 (5.7%)	16 (6.8%)	22 (12%)	0.005	75 (5.8%)	24 (7.6%)	13 (11%)	0.049	
Mother smoked while being pregnant (yes)	179 (10%)	113 (8.6%)	38 (16%)	28 (16%)	<0.001	110 (8.4%)	46 (14%)	23 (21%)	<0.001	
Maternal mental health					<0.001				<0.001	
No depressive or anxious symptoms	1212 (64%)	961 (69%)	143 (52%)	108 (50%)		919 (67%)	222 (61%)	71 (49%)		
High depressive symptoms only	287 (15%)	182 (13%)	60 (22%)	45 (21%)		192 (14%)	67 (19%)	28 (19%)		
High anxious symptoms only	115 (6.1%)	82 (5.9%)	23 (8.4%)	10 (4.7%)		83 (6.0%)	23 (6.4%)	9 (6.2%)		
Comorbid high symptoms	273 (14%)	172 (12%)	49 (18%)	52 (24%)		187 (14%)	49 (14%)	37 (26%)		
Individual ACEs										
Material deprivation										
Out of home placement										
Child protective services										
Conflict with parents										
Tension between parents										
Physical abuse										
Parental separation/divorce										
Death of a parent										
Low SES of household										

Abbreviations: ACEs, adverse childhood experiences; SES, socioeconomic status.

^a^Mean (SD); *n* (%).

^b^Fisher's exact test; Pearson's chi-squared test.

**Table 2:** Associations between cumulative ACE score and prenatal mental health outcomes in the French EDEN cohort (*n* = 1887)

	High depressive symptoms only		High anxious symptoms only		Comorbid high symptoms	
Cumulative ACE Score	OR (95% CI)	aOR (95% CI)^a^	OR (95% CI)	aOR (95% CI)^a^	OR (95% CI)	aOR (95% CI)^a^
0	—	—	—	—	—	—
1	**1.49 (1.05, 2.12)**	**1.48 (1.04, 2.11)**	1.38 (0.84, 2.27)	1.38 (0.83, 2.28)	1.28 (0.88, 1.86)	1.23 (0.84, 1.80)
2	**2.97 (2.03, 4.34)**	**2.82 (1.92, 4.16)**	1.58 (0.84, 2.97)	1.50 (0.80, 2.85)	**2.25 (1.48, 3.41)**	**2.07 (1.35, 3.16)**
≥3	**2.01 (1.33, 3.05)**	**1.81 (1.18, 2.78)**	1.28 (0.65, 2.49)	1.05 (0.53, 2.11)	**2.87 (1.96, 4.20)**	**2.31 (1.55, 3.46)**

*Note:* In bold, statistically significant associations (*p* < 0.05).

Abbrevitions: aOR, adjusted odds ratios; CI, confidence interval; OR, odds ratio.

^a^Multinomial regressions adjusting for age, migrant status, educational level, study center, prematurity, and mother smoked during pregnancy.

**Table 3:** Associations between latent classes of adversity and prenatal mental health outcomes in the French EDEN cohort (*n* = 1887)

	High depressive symptoms only		High anxious symptoms only		Comorbid high symptoms	
Latent classes	OR (95% CI)	aOR (95% CI)^a^	OR (95% CI)	aOR (95% CI)^a^	OR (95% CI)	aOR (95% CI)^a^
Low risk for adversity	—	—	—	—	—	—
Multidimensional adversity	1.63 (0.85, 3.10)	1.34 (0.68, 2.65)	1.12 (0.39, 3.20)	0.80 (0.27, 2.40)	**2.59 (1.47, 4.58)**	1.79 (0.97, 3.32)
Family discordance	**2.13 (1.53, 2.96)**	**2.01 (1.44, 2.81)**	1.26 (0.73, 2.18)	1.19 (0.68, 2.06)	**2.21 (1.58, 3.09)**	**2.04 (1.44, 2.87)**

*Note*: In bold, statistically significant associations (*p* < 0.05).

Abbreviations: CI, confidence interval; OR, odds ratio.

^a^Multinomial regressions adjusting for age, migrant status, educational level, study center, prematurity, and mother smoked during pregnancy.

**Table 4:** Associations between dimensions of adversity and prenatal mental health outcomes in the French EDEN cohort (*n* = 1887)

	High depressive symptoms only			High anxious symptoms only			Comorbid high symptoms		
Dimensions of adversity	OR (95% CI)	aOR (95% CI)^a^	aOR (95% CI)^b^	OR (95% CI)	aOR (95% CI)^a^	aOR (95% CI)^b^	OR (95% CI)	aOR (95% CI)^a^	aOR (95% CI)^b^
Threat score	**1.60 (1.34, 1.91)**	**1.55 (1.29, 1.86)**	**1.50 (1.23, 1.82)**	1.21 (0.91, 1.60)	1.15 (0.86, 1.54)	1.15 (0.84, 1.57)	**1.70 (1.42, 2.03)**	**1.59 (1.32, 1.91)**	**1.57 (1.29, 1.92)**
Deprivation score	**1.39 (1.14, 1.69)**	**1.31 (1.06, 1.61)**	1.10 (0.88, 1.38)	1.17 (0.86, 1.59)	1.07 (0.77, 1.47)	1.01 (0.71, 1.43)	**1.45 (1.19, 1.77)**	**1.26 (1.02, 1.56)**	1.03 (0.82, 1.30)

*Note:* In bold, statistically significant associations (*p* < 0.05).

Abbreviations: aOR, adjusted odds ratios; CI, confidence interval; OR, odds ratio.

^a^Multinomial regressions adjusting for age, migrant status, primary education only, study center, prematurity, and mother smoked during pregnancy.

^b^Multinomial regressions adjusting for age, migrant status, primary education only, study center, prematurity, mother smoked during pregnancy, deprivation score (for threat as main exposure), and threat score (for deprivation as main exposure).

## Data Availability

The data that support the findings of this study are available from the EDEN Consortium but restrictions apply to their availability, which were used under license for the current study and so are not publicly available. Data are, however, available from the authors upon reasonable request and with permission of the EDEN consortium.

## Supporting Information

Supporting Information Table S1. Fit statistics (G2, df, AIC, BIC, aBIC, entropy, and BLRT) used to determine the optimal number of latent classes in the latent class analysis. The three-class model (highlighted in bold) was selected as the best fitting solution. Table S2. The number and percentage of missing data for each variable of interest. Table S3. Characteristics of the participants described by prenatal depression and anxiety symptoms. Table S4. Comparison of characteristics between the included and excluded participants. Table S5. Multinomial logistic regression analyses (crude and adjusted) using multiple imputed datasets to examine associations between individual ACEs and prenatal depression and anxiety symptoms. Table S6. Sensitivity analyses using complete cases. Multinomial logistic regression results are presented for cumulative ACE scores, latent classes, and dimensions of adversity and their associations with prenatal depression and anxiety symptoms. Table S7. Multinomial logistic regression results using an alternative cutoff (≥40) for STAI-S to define high anxiety symptoms.
