# Supplementary material for: Associations Between Adverse Childhood Experiences and Prenatal Mental Health in the French EDEN Cohort: Cumulative, Person-Centered, and Dimensional Approaches
Source: Depress Anxiety. 2025 May 6;2025:1295206. doi: 10.1155/da/1295206 (PMC12074855; doi:10.1155/da/1295206)
Supplement: Supporting Information — Table S1. Fit statistics (G2, df, AIC, BIC, aBIC, entropy, and BLRT) used to determine the optimal number of latent classes in the latent class analysis. The three-class model (highlighted in bold) was selected as the best fitting solution. Table S2. The number and percentage of missing data for each variable of interest. Table S3. Characteristics of the participants described by prenatal depression and anxiety symptoms. Table S4. Comparison of characteristics between the included and excluded participants. Table S5. Multinomial logistic regression analyses (crude and adjusted) using multiple imputed datasets to examine associations between individual ACEs and prenatal depression and anxiety symptoms. Table S6. Sensitivity analyses using complete cases. Multinomial logistic regression results are presented for cumulative ACE scores, latent classes, and dimensions of adversity and their associations with prenatal depression and anxiety symptoms. Table S7. Multinomial logistic regression results using an alternative cutoff (≥40) for STAI-S to define high anxiety symptoms. [file 1295206.f1.docx]

**Supplementary material**

| **Supplementary table 1. Fit statistics for model selection in latent class analysis** | | | | | | | |
| --- | --- | --- | --- | --- | --- | --- | --- |
| **Model** | **G2** | **df** | **AIC** | **BIC** | **aBIC** | **Entropy** | **BLRT** |
| 1-class | 1648.47 | 502 | 1666.47 | 1716.35 | 1716.35 | 1.00 | - |
| 2-class | 519.71 | 492 | 557.71 | 663.03 | 602.66 | 0.82 | <0.001 |
| **3-class** | **382.25** | **482** | **440.25** | **600.99** | **508.85** | **0.84** | **<0.001** |
| 4-class | 288.40 | 472 | 366.40 | 582.57 | 458.67 | 0.88 | <0.001 |

G2: Goodness-of-fit statistics; df: degrees of freedom; AIC: Akaike’s information criteria; BIC: Bayesian information criteria; aBIC: adjusted Bayesian information criteria; BLRT: Bootstrapped likelihood ratio test; In bold, the selected solution for latent class analysis

| **Supplementary table 2. Missing data on variables of interest** | |
| --- | --- |
| **Characteristics** | **Missing data**^a^ |
| Age (in years) | 17 (0.90) |
| Primary education only (yes) | 24 (1.27) |
| Migration status | 29 (1.54) |
| Study center (Poitiers) | 0 |
| Personal history of prematurity (yes) | 164 (8.69) |
| Mother smoked while being pregnant (yes) | 146 (7.74) |
| Material deprivation | 0 |
| Out of home placement | 1 (0.05) |
| Child protective services | 8 (0.42) |
| Conflict with parents | 9 (0.48) |
| Tension between parents | 18 (0.95) |
| Physical abuse | 6 (0.32) |
| Parental separation/divorce | 2 (0.11) |
| Death of a parent | 18 (0.95) |
| Low SES of household | 15 (0.79) |
| **^a^ n(%)** | |

| **Supplementary table 3. Sample characteristics of EDEN participants by prenatal depression and anxiety symptoms**   \| **Characteristic** \| **N** \| **Anxious symptoms only**  N = 115^a^ \| **Comorbid symptoms**  N = 273*^a^* \| **Depressive symptoms only** N = 287*^a^* \| **Non depressive, non anxious**  N = 1,212*^a^* \| **p-value***^b^* \| \| --- \| --- \| --- \| --- \| --- \| --- \| --- \| \| Age (in years) \| 1,870 \| 29.65 (5.03) \| 29.49 (5.25) \| 29.23 (5.14) \| 29.63 (4.68) \| 0.5 \| \| Primary education only (yes) \| 1,863 \| 15 (13%) \| 38 (14%) \| 18 (6.4%) \| 59 (4.9%) \| <0.001 \| \| Migration status \| 1,858 \|  \|  \|  \|  \| <0.001 \| \| None \|  \| 98 (88%) \| 207 (77%) \| 237 (83%) \| 1,051 (88%) \|  \| \| 2nd generation \|  \| 10 (8.9%) \| 41 (15%) \| 33 (12%) \| 108 (9.1%) \|  \| \| 1st generation \|  \| 4 (3.6%) \| 21 (7.8%) \| 15 (5.3%) \| 33 (2.8%) \|  \| \| Study center (Poitiers) \| 1,887 \| 65 (57%) \| 143 (52%) \| 126 (44%) \| 587 (48%) \| 0.073 \| \| Personal history of prematurity (yes) \| 1,723 \| 6 (5.9%) \| 14 (6.0%) \| 29 (11%) \| 63 (5.6%) \| 0.007 \| \| Mother smoked while being pregnant (yes) \| 1,741 \| 9 (8.3%) \| 31 (13%) \| 37 (14%) \| 102 (9.0%) \| 0.042 \| \| *^a^* Mean (SD); n (%); *^b^* Kruskal-Wallis rank sum test; Pearson's Chi-squared test \| \| \| \| \| \| \| |
| --- | --- | --- | --- | --- | --- | --- | --- | --- | --- | --- | --- | --- | --- | --- | --- | --- | --- | --- | --- | --- | --- | --- | --- | --- | --- | --- | --- | --- | --- | --- | --- | --- | --- | --- | --- | --- | --- | --- | --- | --- | --- | --- | --- | --- | --- | --- | --- | --- | --- | --- | --- | --- | --- | --- | --- | --- | --- | --- | --- | --- | --- | --- | --- | --- | --- | --- | --- | --- | --- | --- | --- | --- | --- | --- | --- | --- | --- |

| **Supplementary table 4. Characteristics of included and excluded participants in the study sample** | | | | |
| --- | --- | --- | --- | --- |
| **Variable** | **N** | **Excluded**  N = 115^a^ | **Included**  N = 1,887^a^ | **p-value^b^** |
| Age (in years) | 1,907 | 26.0 (5.4) | 29.6 (4.9) | <0.001 |
| Primary education only (yes) | 1,892 | 13 (15%) | 130 (7.0%) | <0.001 |
| Mother smoked while being pregnant (yes) | 1,825 | 12 (14%) | 179 (10%) | 0.2 |
| Personal history of prematurity (yes) | 1,803 | 3 (3.8%) | 112 (6.5%) | 0.3 |
| Migration status | 1,887 |  |  | 0.028 |
| None |  | 21 (72%) | 1,593 (86%) |  |
| Second generation |  | 4 (14%) | 192 (10%) |  |
| First generation |  | 4 (14%) | 73 (3.9%) |  |
| Study center (Poitiers) | 2,002 | 67 (58%) | 966 (51%) | 0.14 |
| ^a^Mean (SD) ; n (%); ^b^Fisher's exact test; Pearson's Chi-squared test | | | | |
|  | | | | |

Supplementary table 5. Associations between individual ACEs and prenatal depressive and anxious symptoms using multiple imputed data (n=1887)

|  | **High Symptoms of Depression Only** | | **High Symptoms of Anxiety Only** | | **Comorbid Symptoms** | |
| --- | --- | --- | --- | --- | --- | --- |
| **ACEs** | **OR (95% CI)** | **aOR (95% CI)**^a^ | **OR (95% CI)** | **aOR (95% CI)**^a^ | **OR (95% CI)** | **aOR (95% CI)**^a^ |
| Material deprivation | 1.55 (0.99, 2.44) | 1.08 (0.65, 1.77) | 1.07 (0.50, 2.28) | 0.85 (0.38, 1.91) | **2.39 (1.59, 3.60)** | 1.42 (0.89, 2.27) |
| Out of home placement | 1.69 (0.83, 3.43) | 0.97 (0.40, 2.38) | 1.54 (0.53, 4.46) | 1.10 (0.27, 4.46) | **2.29 (1.19, 4.40)** | 1.01 (0.43, 2.39) |
| Child protective services | 1.54 (0.71, 3.34) | 0.75 (0.28, 1.99) | 1.70 (0.58, 4.98) | 1.02 (0.25, 4.21) | 1.99 (0.97, 4.10) | 0.64 (0.25, 1.67) |
| Subjected to abuse or beatings | **1.86 (1.18, 2.92)** | 1.15 (0.67, 1.97) | 0.91 (0.39, 2.14) | 0.61 (0.23, 1.58) | **2.61 (1.71, 3.99)** | 1.43 (0.85, 2.39) |
| Tension or violence between parents | **2.03 (1.51, 2.73)** | **1.72 (1.20, 2.47)** | 1.41 (0.89, 2.24) | 1.43 (0.83, 2.47) | **2.22 (1.65, 2.99)** | **1.93 (1.34, 2.77)** |
| Conflict with parents | **2.00 (1.40, 2.87)** | 1.32 (0.84, 2.08) | 1.19 (0.65, 2.18) | 1.12 (0.55, 2.31) | **2.05 (1.42, 2.94)** | 1.19 (0.75, 1.90) |
| Death of a parent | 1.50 (0.77, 2.94) | 1.36 (0.66, 2.81) | 1.26 (0.44, 3.62) | 1.12 (0.37, 3.40) | **2.04 (1.09, 3.79)** | 1.52 (0.76, 3.04) |
| Parental separation/ divorce | 1.27 (0.91, 1.79) | 0.88 (0.60, 1.28) | 1.06 (0.63, 1.80) | 0.91 (0.51, 1.63) | 0.90 (0.61, 1.31) | **0.56 (0.36, 0.86)** |
| Low SES of household | 1.66 (0.95, 2.91) | 1.41 (0.76, 2.62) | 1.38 (0.58, 3.31) | 1.34 (0.55, 3.29) | **1.99 (1.16, 3.42)** | 1.42 (0.76, 2.65) |
| Multinomial regressions adjusting for: age, migrant status, educational level, study center, prematurity, mother smoked during pregnancy. In bold, statistically significant associations (p<0.05); CI: confidence interval; OR: odds ratio | | | | | | |

| Supplementary table 6. Associations between ACEs and prenatal mental health outcomes in the French EDEN cohort using complete cases (n=1587) | | | | | | | |
| --- | --- | --- | --- | --- | --- | --- | --- |
|  | **High Symptoms of Depression Only** | | **High Symptoms of Anxiety Only** | | **Comorbid Symptoms** | | |
| **Cumulative ACEs Score** | **aOR (95% CI)**^a^ | | **aOR (95% CI)**^a^ | | **aOR (95% CI)**^a^ | | |
| 0 | — | | — | | — | | |
| 1 | 1.37 (0.93, 2.02) | | 1.56 (0.91, 2.68) | | 1.07 (0.69, 1.66) | | |
| 2 | **2.48 (1.58, 3.87)** | | 1.36 (0.62, 2.97) | | **2.12 (1.31, 3.44)** | | |
| ≥3 | **1.84 (1.12, 3.02)** | | 1.44 (0.67, 3.11) | | **2.65 (1.67, 4.21)** | | |
| **Latent classes** | | | | | | | |
| Low risk for adversity | — | | — | | — | | |
| Multidimensional adversity | 1.15 (0.47, 2.82) | | 1.15 (0.32, 4.12) | | 1.86 (0.83, 4.16) | | |
| Family discordance | **1.84 (1.25, 2.70)** | | 1.18 (0.62, 2.25) | | **2.22 (1.51, 3.27)** | | |
| **Dimensions of adversity** | **aOR (95% CI)**^a^ | **aOR (95% CI)^b^** | **aOR (95% CI)**^a^ | **aOR (95% CI)^b^** | **aOR (95% CI)**^a^ | **aOR (95% CI)^b^** | |
| Threat score | **1.48 (1.21, 1.82)** | **1.43 (1.14, 1.78)** | 1.28 (0.93, 1.75) | 1.26 (0.90, 1.78) | **1.69 (1.37, 2.07)** | **1.68 (1.35, 2.09)** | |
| Deprivation score | **1.31 (1.03, 1.67)** | 1.14 (0.87, 1.48) | 1.14 (0.78, 1.65) | 1.03 (0.69, 1.55) | 1.27 (0.99, 1.64) | 1.01 (0.77, 1.34) | |
| ^a^Multinomial regressions adjusting for: age, migrant status, primary education only, study center, prematurity, mother smoked during pregnancy; ^b^Multinomial regressions adjusting for: age, migrant status, primary education only, study center, prematurity, mother smoked during pregnancy, deprivation score (for threat as main exposure), threat score (for deprivation as main exposure); In bold, statistically significant associations (p<0.05); CI: confidence interval; OR: odds ratio. | | | | | | |  |

| **Supplementary table 7. Associations between ACEs and prenatal mental health outcomes in the French EDEN cohort using ≥40 *as* alternative cut-off for STATI-S (n=1887)** | | | | | | | | | | | | | | | | | |
| --- | --- | --- | --- | --- | --- | --- | --- | --- | --- | --- | --- | --- | --- | --- | --- | --- | --- |
| **Cumulative ACEs Score** | **High Symptoms of Depression Only**  **n(%) = 328(17.4%)** | | | | | **High Symptoms of Anxiety Only**  **n(%) = 88(4.7%)** | | | | | | **Comorbid Symptoms**  **n(%) = 232(12.3%)** | | | | | |
|  | **OR (95% CI)** | | **aOR (95% CI)** | | | **OR (95% CI)** | | | **aOR (95% CI)** | | | **OR (95% CI)** | | | **aOR (95% CI)** | | |
| 0 | — | | — | | | — | | | — | | | — | | | — | | |
| 1 | **1.40 (1.00, 1.95)** | | 1.39 (0.99, 1.94) | | | 1.05 (0.58, 1.89) | | | 1.05 (0.58, 1.90) | | | 1.29 (0.87, 1.92) | | | 1.23 (0.82, 1.84) | | |
| 2 | **2.81 (1.96, 4.04)** | | **2.69 (1.86, 3.89)** | | | 1.43 (0.71, 2.89) | | | 1.39 (0.68, 2.83) | | | **2.16 (1.38, 3.37)** | | | **1.97 (1.25, 3.12)** | | |
| ≥3 | **1.95 (1.32, 2.88)** | | **1.77 (1.18, 2.65)** | | | 0.80 (0.34, 1.90) | | | 0.66 (0.27, 1.61) | | | **3.00 (2.02, 4.45)** | | | **2.37 (1.55, 3.61)** | | |
| **Latent classes** |  | | |  | | |  | | |  | | |  | | |  | |
| Low risk for adversity | — | | — | | | — | | | — | | | — | | | — | | |
| Multidimensional adversity | 1.70 (0.94, 3.08) | | 1.43 (0.76, 2.67) | | | 0.69 (0.16, 2.91) | | | 0.49 (0.11, 2.19) | | | **2.50 (1.37, 4.57)** | | | 1.69 (0.88, 3.24) | | |
| Family discordance | **2.06 (1.51, 2.82)** | | **1.96 (1.43, 2.70)** | | | 1.12 (0.59, 2.10) | | | 1.07 (0.56, 2.02) | | | **2.24 (1.57, 3.19)** | | | **2.05 (1.43, 2.95)** | | |
| **Dimensions of adversity** | **OR (95% CI)** | **aOR (95% CI)^a^** | | | **aOR (95% CI)^b^** | **OR (95% CI)** | | **aOR (95% CI)^a^** | | | **aOR (95% CI)^b^** | **OR (95% CI)** | | **aOR (95% CI)^a^** | | | **aOR (95% CI)^b^** |
| Threat score | **1.58 (1.33, 1.86)** | **1.53 (1.29, 1.82)** | | | **1.50 (1.24, 1.80)** | 1.11 (0.79, 1.54) | | 1.06 (0.75, 1.49) | | | 1.14 (0.79, 1.64) | **1.70 (1.41, 2.05)** | | **1.59 (1.31, 1.93)** | | | **1.58 (1.28, 1.95)** |
| Deprivation score | **1.35 (1.12, 1.62)** | **1.28 (1.05, 1.55)** | | | 1.08 (0.87, 1.33) | 0.93 (0.64, 1.37) | | 0.86 (0.58, 1.27) | | | 0.82 (0.53, 1.25) | **1.46 (1.18, 1.80)** | | **1.25 (1.00, 1.56)** | | | 1.02 (0.80, 1.30) |
| ^a^Multinomial regressions adjusting for: age, migrant status, primary education only, study center, prematurity, mother smoked during pregnancy; ^b^Multinomial regressions adjusting for: age, migrant status, primary education only, study center, prematurity, mother smoked during pregnancy, deprivation score (for threat as main exposure), threat score (for deprivation as main exposure); In bold, statistically significant associations (p<0.05); CI: confidence interval; OR: odds ratio. | | | | | | | | | | | | | | | | | |
